# Human papillomavirus vaccination in low- and middle-income countries: progression, barriers, and future prospective

**DOI:** 10.3389/fimmu.2023.1150238

**Published:** 2023-05-12

**Authors:** Narges Ebrahimi, Zahra Yousefi, Gholamreza Khosravi, Fatemeh Eskandari Malayeri, Marjan Golabi, Monireh Askarzadeh, Mohammad Hossein Shams, Behrooz Ghezelbash, Nahid Eskandari

**Affiliations:** ^1^ Immunology Department, Faculty of Medicine, Isfahan University of Medical Sciences, Isfahan, Iran; ^2^ School of Allied Medical Sciences, Shahroud University of Medical Sciences, Shahroud, Iran; ^3^ Department of Medical Immunology, School of Medicine, Lorestan University of Medical Sciences, Khorramabad, Iran

**Keywords:** human papilloma virus (HPV), vaccination, low-and middle-income countries (LMICs), cervical cancer, immunization program

## Abstract

Human papillomavirus (HPV) is a viral infection that, if does not go away, can cause health problems like genital warts and cancer. The national immunization schedules for individuals before sexual debut, significantly decreased HPV-associated mortality and it will be affordable. However, immunization programs remain vulnerable to macroeconomic factors such as inflation, fiscal policy, employment levels, and national income. This review aims to investigate the association between national income in lower-middle-income countries to explore recent advances and potential issues, as well as how to deal with challenges.

## 1 Introduction

Papillomaviruses are epitheliotropic, small, uncoated, double-stranded DNA viruses that contaminate mucosal and dermal epithelium in a broad diversity of high vertebrates. Human papillomavirus (HPV), with a global prevalence of 11.7%, is the cause of one of the most common sexually transferred diseases worldwide (1, 2). It is known that approximately 70% of sexually active people will be infected with the HPV virus at least once in their lifetime (3–5). The highest rate of HPV infection is seen among women from 16 to 25 years old. Approximately 70% of HPV infections are automatically removed after several months. However, the virus’s continuance could lead to changes in cell growth and cervical cancer initiation (6).

**Figure 1:**
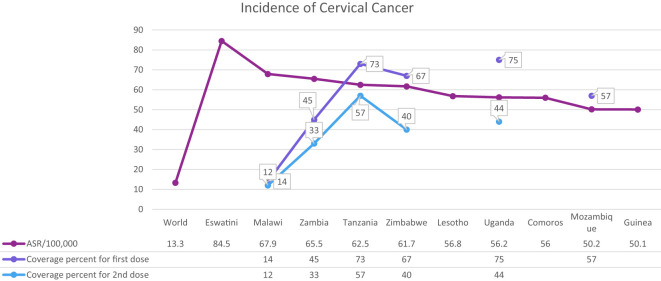
Global cervical cancer incidence in 2020 and HPV coverage percent in each country.

**Figure 2:**
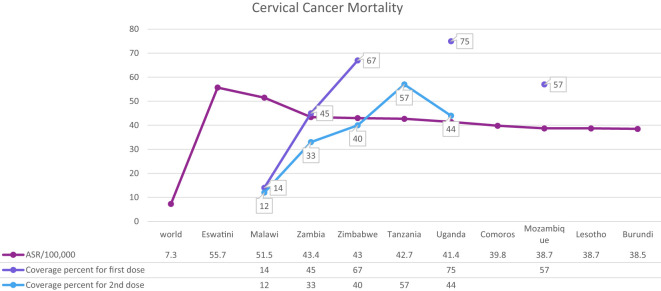
Global mortality rate caused by cervical cancer and HPV coverage percent in each country.

Cervical cancer is the fourth most common cancer among women in the world and especially in low and middle-income countries (LMICs) such as Brazil, India, China, South Africa (SA), and Iran (7–9) (**Figure 1**). HPV 16 and 18 are the most common subtypes of HPV, which cause 70% of cervical cancer patients and are also associated with tumors of the anus, vulva, and penis (10). Overall ~569,000 new patients with cervical cancer and 311,000 deaths related to this cancer were reported worldwide in 2018 (**Figure 2**). Although HPV infections and their related malignancies are common in areas with high socioeconomic status, on the whole, 84% of new patients with cervical cancer and between 87% to 90% of the dead women from this cancer occurred in LMICs, which have little ability to perform extensive national screening and pre-cancer therapy schedules (7, 9, 11, 12) and thousands of women’s die prematurely from cervical cancer in LMICs than developed countries (13, 14).

HPV immunization could control nearly 70% of all cervical cancers (15) (**Tables 1**, **2**). Prevention and management of cervical cancer are performed by two processes, including pap screening for early cancer diagnosis and vaccination against HPV to prevent cervical cancer progression (13). There are four vaccines against HPV: the quadrivalent (4vHPV) Gardasil, bivalent (2vHPV) Cervarix, 9-valent (9vHPV) Gardasil-9 and the bivalent Cecolin in China (1, 16). The U.S.FDA approved Gardasil 4 for precluding anogenital cancer, and genital warts use in females and males in 2006 and 2009, in order and Gardasil 9 in 2015 (17).

**Table 1:** Cervical cancer rates 2020; this table shows global cervical cancer incidence in 2020.

Rank	Country	Number	ASR/100,000	Target for first dose of HPV	Target for first dose of HPV	Coverage percent for first dose	Target for second dose	2nd dose HPV immunization completion	Coverage percent for 2nd dose
	**World**	**604,127**	**13.3**	NA	NA	NA	NA	NA	NA
**1**	Eswatini	341	84.5	NA	NA	NA	NA	NA	NA
**2**	Malawi	4,145	67.9	268,574	36,402	14	265,988	32,356	12
**3**	Zambia	3,161	65.5	235,232	106,398	45	229,792	76,384	33
**4**	Tanzania	10,241	62.5	735,771	535,298	73	735,771	420,015	57
**5**	Zimbabwe	3,043	61.7	415,769	27,809	67	417,569	165,532	40
**6**	Lesotho	541	56.8	NA	NA	NA	NA	NA	NA
**7**	Uganda	6,959	56.2	666,329	499,667	75	666,329	293,699	44
**8**	Comoros	167	56	NA	NA	NA	NA	NA	NA
**9**	Mozambique	5,325	50.2	443,702	25,1124	57	NA	NA	NA
**10**	Guinea	2,068	50.1	NA	NA	NA	NA	NA	NA

Eswatini (which changed its name from Swaziland in 2018) had the highest rate of cervical cancer in 2020, followed by Malawi. www.wcrf.org & www.who.int.

NA, Not applicable.

**Table 2:** Global cervical cancer mortality in 2020.

Rank	Country	Number	ASR/100,000	Target for first dose of HPV	Target for first dose of HPV	Coverage percent for first dose	Target for second dose	2nd dose HPV immunization completion	Coverage percent for 2nd dose
	**World**	**341,831**	**7.3**	NA	NA	NA	NA	NA	NA
**1**	Eswatini	214	55.7	NA	NA	NA	NA	NA	NA
**2**	Malawi	2,905	51.5	268,574	36,402	14	265,988	32,356	12
**3**	Zambia	1,904	43.4	235,232	106,398	45	229,792	76,384	33
**4**	Zimbabwe	1,976	43	417,569	278,070	67	417,569	165,532	40
**5**	Tanzania	6,525	42.7	NA	NA	NA	735,771	420,015	57
**6**	Uganda	4,607	41.4	666,329	499,667	75	666,329	293,699	44
**7**	Comoros	109	39.8	NA	NA	NA	NA	NA	NA
**8**	Mozambique	3,850	38.7	443,702	251,124	57	NA	NA	NA
**9**	Lesotho	362	38.7	NA	NA	NA	NA	NA	NA
**10**	Burundi	1,126	38.5	NA	NA	NA	NA	NA	NA

Eswatini had the highest rate of cervical cancer mortality in 2020, followed by Malawi. www.wcrf.org & www.who.int.

NA, Not applicable.

Due to girls aged 9 to 14 not yet sexually involved, World Health Organization (WHO) suggested these ages as the most suitable time for vaccination against HPV (18). The national immunization schedules for young women before sexual activity significantly decreased HPV-associated mortality and will be affordable (19). The HPV vaccine is affordable in multiple countries. Ninety-six countries have used the HPV vaccine in their national immunization course (2). However, the advancement of LMIC toward decreasing the load of cervical cancer is prolonged (20). This is due to several reasons, including slow expansion of HPV vaccination, low rates of screening and early diagnosis of cervical cancer, restricted availability of exhaustive cancer therapy, price, problem in successfully acquiring HPV vaccine target people, cultural matters connected to the HPV vaccine rumors, little understanding of cervical cancer and its association to HPV infection, troubles about HPV vaccination considering future safety and fertility, negative background with prior vaccinations for other illnesses, and political issues (21–28). Unlike high-income countries (HICs), smaller than 30% of LMICs have presented the program of HPV vaccination and approximately 3% of teenagers were vaccinated against HPV, and about 44% of women were screened for cervical cancer (20). A three-dose schedule of HPV vaccination for teens and young adults was recommended by WHO in 2009 (29), and support of LMICs to perform the immunization program was confirmed by Gavi, the Vaccine Alliance, in 2011. Based on updated proof, the WHO edition of this program in 2014 suggested a 2-dose regimen for girls 9 to 14 years old (30). This program was edited in 2017 to indicate that governments should implement multi-age cohorts (MAC) rather than a single cohort for vaccination. Early vaccine presentation could increase the effect and efficiency of the vaccination schedule (29). With restrained HPV vaccine supply becoming apparent in 2018, the Strategic Advisory Group of Experts on Immunization (SAGE) suggested 2019 an interim break in MAC vaccinations and even entertained the feasibility of a long gap of 2–3 years between the first and second doses of vaccine (16). In 2020, WHO determined a world strategy to speed up the management of cervical cancer and immunize about 90% of 15-year-old girls (31).

However, according to the most current research, which was published in December 2022, WHO recommends: a one or two-dose schedule for girls aged 9 to 14 years; a one or two-dose schedule for girls and women aged 15 to 20 years; and two doses with a 6-month interval for women over 21 years.

WHO recommended that HPV vaccination be incorporated into national immunization schedules for the preliminary target individuals of girls 9 to 14 and the secondary target individuals of women over 15 years of age (29). In mid-2020, 56 LMICs (41% of all LMICs) initiated national HPV vaccine schedules and were approved by Gavi (from May 2020) to the availability of resources. The number of countries that use these vaccination programs has increased each month. Millions of girls in developing countries are protected against cervical cancer thanks to new HPV vaccine deals. 2013 (32). It has been identified that a high range of HPV vaccination in lassies can decrease cervical cancer in most LMICs until the end of the century (33). Nevertheless, in the lack of more endeavors to enhance vaccination coverage, 44.4 million patients with cervical cancer will be recognized worldwide over the years 2020–69, with nearly two-thirds of the patients happening in nations with low-Human Development Indices (34).

Collectively, the timely, accurate introduction and implementation of HPV vaccination in national programs is an essential step in eliminating cancer and other diseases caused by HPV. Recent developments in this field are promising, although still associated with challenges. Hence, this study reviews different aspects of HPV vaccination status in low- and middle-income countries.

## 2 Progression of HPV vaccine

**Figure 3:**
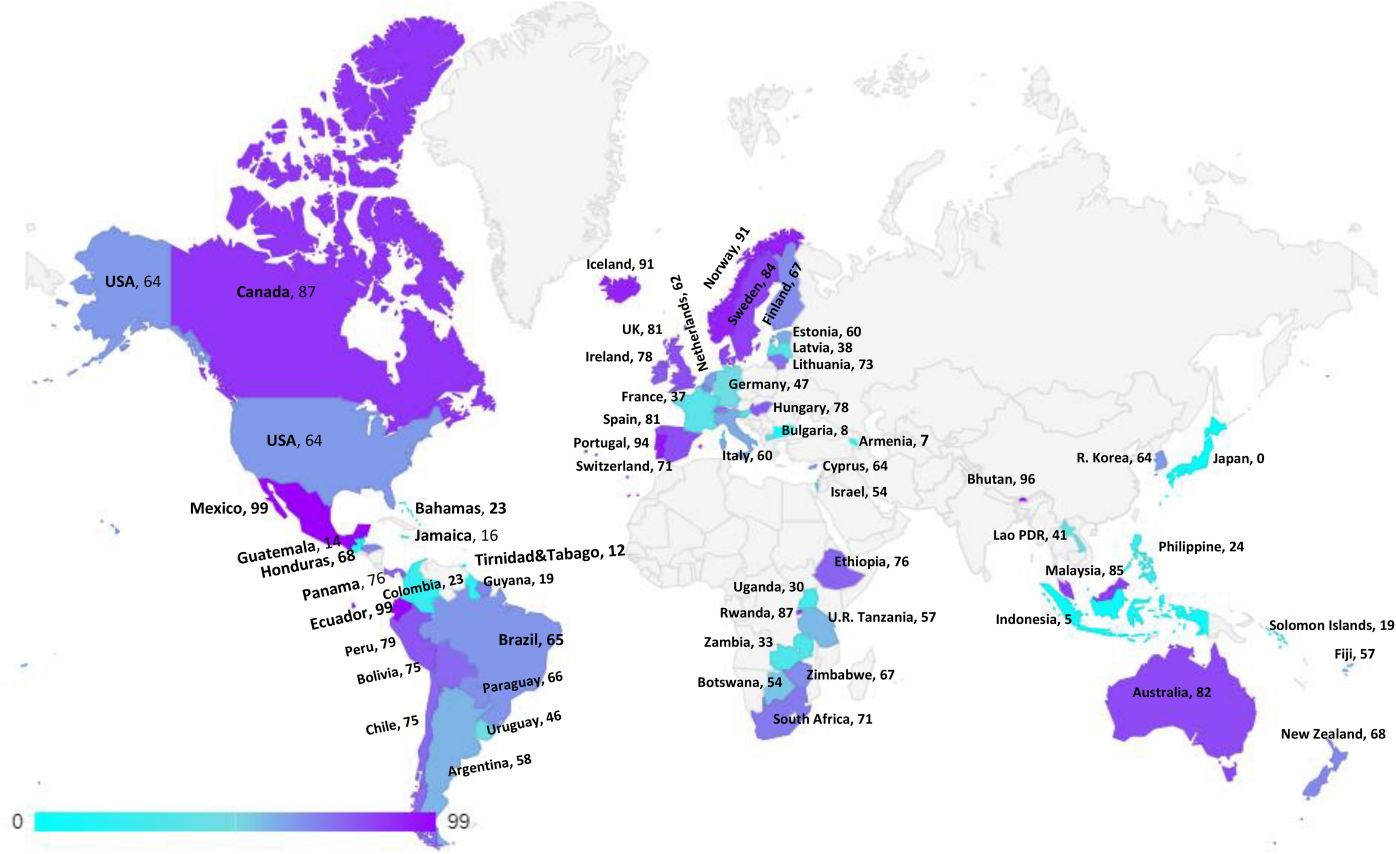
HPV Vaccination coverage by age 15, last dose, females low, middle and high income countries.

Besides the progress and challenges of the LMIC vaccination introduction that will be outlined in this study, the achievements of HPV immunization in high-income countries could be a helpful roadmap for developing nations (**Figure 3**). The ongoing in developed nations could be tracked in some of the European countries and Australia, which have achieved high immunization and screening coverage in the target women population, in addition to shot recommendations for adolescent males in some regions (35). One of the successful pioneers in the European Union is Belgium’s Flanders region, with a vaccination uptake rate of 91%. The factors contributing to high coverage in this community compared to other regions with lower success are raised vaccine awareness among healthcare providers and parents, less influence of media rumors from neighboring communities, and a more thorough vaccination program *via* School Health Services (SHS) with enhanced vaccine databases (36). In Sweden, the vaccination coverage has reached over 80%, since the introduction of the school-based program with fully-funded quadrivalent vaccine offers in 2010 (37). This mode of vaccine delivery showed the highest efficiency in increasing uptakes and reducing social disparities related to the program in a nationwide cohort study (38). Although there is vaccine hesitancy in communities with lower education and socioeconomic status, there is a substantial positive attitude toward vaccination in a recent survey in Sweden with a rate of 93%, highlighting the influence of providing health information and healthcare providers’ role in discussing vaccine concerns with parents (37).

Regarding Australia and its great success in implementing the national HPV immunization program, the evidence indicates that its vaccine coverage is among the highest rates globally regardless of gender, while in 2017, the rate was over 89% and 86% in girls and boys at age 15 for the single dose, respectively (39). The country initiated its national program in 2007, with a 3-dose quadrivalent vaccine offered through highly efficient government-funded school-based delivery. This program also involved multi-age cohorts with a broader age range, including a free catch-up program for women 18 to 26 years of age (40) and a primary care vaccine offer for those who missed the school-based program up to age 19 (41). Despite some obstacles and uptake variations in on-site school-based vaccine delivery, such as schools with small sizes and higher attendance of indigenous students, this approach remains one of the most efficacious delivery strategies in Australia (39). The country transitioned to a 2-dose nonavalent vaccine in 2018 and is close to achieving 90% coverage by the school-based program that could enhance herd immunity for unvaccinated women and new immigrants (41). The nonavalent vaccine introduction could enhance the lowering of HPV-related cancers by preventing over 90% of cervical and anal cancers (42). Keeping these vaccination trends and screening programs, Australia is predicted to reduce the cervical cancer rate to less than 4 cases per 100000 women per year by 2028 and possibly be the first nation to eliminate cervical cancer by 2040 (34, 43).

### 2.1 Target population and effective strategies for HPV vaccination

HPV vaccination began in 2006 and has been recommended by WHO since 2009. HPV vaccines have been traditionally introduced in many national immunization schedules, but several studies and international agencies have reported insignificant vaccine introduction and coverage (30, 44, 45).

As of March 2022, 117 countries (60% of WHO member states, approximately one-third of the global target population) have included the HPV vaccine in their routine national immunization schedules. Otherwise, despite accounting for most of the disease burden in LMICs, the adoption rate remains lower than in HICs (46). The standard recommendation is a 2-dose schedule at least six months apart for those under 15 years old at the first dose and a 3-dose schedule (0, 6, 12 months) for those 15 years or older, as well as those with immunodeficiency or HIV infection. Alternative plans with extended dosing intervals are also used in some countries (47, 48). Governments need to take into account some factors when arranging for vaccine distribution, such as the primary and secondary sites for vaccination, the timing, and duration of distribution, and whether the distribution was carried out in conjunction with other health or community activities. In order to increase HPV vaccination rates in schools, it is necessary to implement a comprehensive communication plan that includes professional development for educators, targeted school messages, and the use of mass media. In places employing facility- or community-based solutions, a continuous schedule approach is adopted, with continual messaging or activities from health workers or community agents. Another notification in delivery strategies for the HPV vaccine is its combination with other health or educational services. These approaches have been undertaken by several LMICs with different periods (32, 35, 49–51).

**Figure 4:**
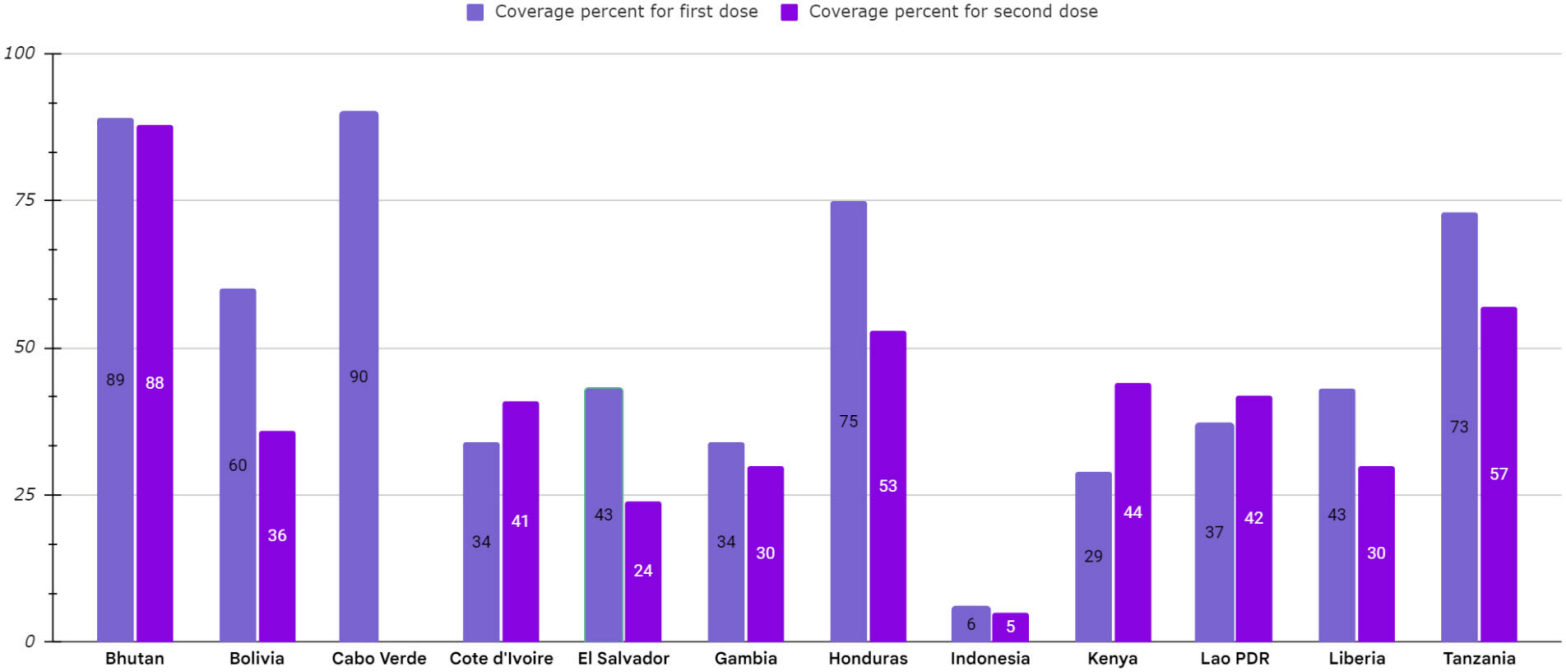
HPV vaccination program coverage, first and second dose, females in low-and middle-income countries.

The most critical problem in HPV vaccination is identifying eligible individuals; otherwise, opportunities for immunization may be missed, and it is beneficial to define narrow criteria for determining the target population. For example, grade-based eligibility can be helpful in school-based strategies in countries with high school attendance and a narrow age spectrum of girls in each class. However, for community or facility-based approaches, or where out of school (OOS) girls constitute a significant proportion of the population, age-based eligibility is usually preferable (32). Usually, younger girls (9–10 years) in LMICs than HICs (11–13 years) are targeted for HPV vaccination (35). As mentioned, the age range of vaccination varies from 9 to 14 years. First-dose coverage is more than second-dose coverage because subjects may be out-of-school when the second dose is needed. As a result, targeted messaging and social media strategies can effectively increase community participation to ensure they receive the second dose of the HPV vaccine (52). However, the primary method for HPV vaccine delivery in LMICs with national HPV vaccination is school-based (Argentina, Armenia, Brazil, Bulgaria, Georgia, Moldova, Turkmenistan) or mixed (Cote d’Ivoire, Ecuador, Kenya, Liberia, Malawi, Mexico, Senegal, Tanzania, Uganda), whereas in LMICs without national HPV vaccination (Haiti, Mozambique, Bangladesh) is usually mixed. There is no comprehensive information available about countries that lack national immunization. Using a combination of delivery strategies, out-of-school girls and populations can receive the HPV vaccine. However, studies have shown that initial coverage is much lower in countries where delivery is based on health facilities, provision of routine immunization, and social mobilization efforts to educate girls and their families than in countries where delivery is based on school-based strategies (35, 49, 53–59). Most LMICs have reported a two-dose HPV vaccination schedule with a 6-month interval. However, some countries (Gambia, Lao, People’s Democratic Republic, Senegal, Solomon Islands, Zimbabwe, Zambia) use a 12-month gap between the first and second dose of the vaccine (**Figure 4**, **Table 3**) (32, 35, 52, 54, 60–69).

**Table 3:** Low-and middle-income countries, HPV vaccination coverage has been compared with Measles vaccine, 2021 https://data.worldbank.org/ & www.who.int.

Country	Total cohort	Female cohort	Target for first dose of HPV	1st dose HPV immunization completion	Coverage percent for first dose	Target for second dose	2nd dose HPV immunization completion	Coverage percent for second dose	Vaccine preventable disease measles	Measles Coverage 1st dose	Measles Coverage 2nd dose
**Bhutan**	779,900	364,949	6433	5733	89	6433	5652	88		97	91
**Bolivia**	11,832,936	5,896,513	114656	69339	60	114656	40951	36	0	75	56
**Cabo Verde**	561,901	279,806	5174	4635	90	NA	NA	NA	0	95	86
**Cote d’Ivoire**	27,053,629	13,420,382	1745712	601359	34	341192	138335	41	1837	68	1
**El Salvador**	6,518,500	3,468,951	95701	41152	43	110987	26334	24	0	86	71
**Gambia**	2,486,937	1,253,561	33737	11340	34	148724	45301	30	0	79	67
**Honduras**	10,062,994	5,034,311	98526	73408	75	98526	52421	53	0	81	75
**Indonesia**	276,361,788	137,230,499	2238149	132922	6	2238149	102603	5	394	72	50
**Kenya**	54,985,702	27,662,874	2984592	877907	29	683804	303435	44	266	89	57
**Lao PDR**	7,379,358	3,675,501	75080	28126	37	373719	157966	42	2	73	50
**Liberia**	5,180,208	2,575,144	65265	28325	43	65265	19368	30	250	58	35
**Tanzania**	61,498,438	30,759,748	735771	535298	73	735771	420015	57	0	NA	NA

In **Table 3**-, low- and middle-income countries based on their HPV vaccination data availability have been summarized however, the full table is available in supplementary.

NA, Not applicable.

### 2.2 Preparation, establishment, and perceived difficulties

HPV vaccination could significantly affect the prevention of cervical cancer (70, 71). For instance, in Rwanda and Bhutan, 90% coverage with the quadrivalent vaccine has dramatically diminished the prevalence of significant types of HPV (HPV-6, -11, -16, and -18) and also led to cross-protecting against other types of HPV (HPV -31, -33, -35, -52, and -58) (64). Some determinants may limit the HPV vaccination schedule. One significant issue is that many LMICs do not have a national HPV vaccination program. There are no data about the coverage of the HPV vaccine in these countries. On the other hand, cost and economic problems around HPV vaccination and lack of sufficient human resources have tremendous adverse effects on HPV vaccination in these countries (19, 54, 55, 57, 64, 72). Apart from discussing economic issues, people’s aversion to HPV vaccination is motivated by various factors. Many studies revealed that refusal to take the HPV vaccine was associated with insufficient knowledge and increasing knowledge levels about the vaccine among healthcare institutions and school staff (52, 54, 55). Furthermore, a cross-sectional study conducted in several Arab countries, including Jordan, Qatar, and the United Arab Emirates (UAE), revealed that younger subjects (18-25 years old), who had postgraduate study, education, or a career in the medical field, and had a pap smear in the previous three years, had greater knowledge and awareness about the HPV vaccine than others. As a result, public awareness of cervical cancer and HPV vaccines is required (53, 54, 57, 73, 74). It is also impossible to educate girls about the HPV vaccine without sex education. Parents opposed vaccination because they believed it would cause implicit motivation for sexual activities. Furthermore, some religious groups have a low tendency to vaccines due to their beliefs, such as God protecting people (55, 75–77). Another reason for unwillingness to the vaccine was bad experiences with different vaccines such as measles, making unpleasant feelings about HPV vaccines (54, 56, 78). Recently the COVID-19 pandemic caused more disruption in immunization programs and low coverage in LMICs (46, 60, 79). These reasons could have negative effects on willingness to vaccination. Therefore, a training program for managing these issues is necessary. It was demonstrated that HPV vaccination, combined with health education by health care providers, school personnel, and parents, resulted in high vaccination coverage and HPV vaccine acceptance (55, 75–77). Multiple studies have shown that explaining vaccine side effects to subjects can increase their willingness to receive the vaccine (57, 80). Also, trusting doctors and their recommendations positively affect vaccine acceptance among subjects (55, 67, 73). Using interactive PowerPoint slides and recorded videos to introduce the HPV vaccine could be helpful, similar during the COVID-19 pandemic. Currently, 13% of girls worldwide are protected against HPV, and the global supply is sufficient to meet the global demand. However, a shortage of supply buffers may affect product access over the next three years. The new HPV vaccine Cecolin is a bivalent HPV vaccine manufactured by Innovax and prequalified by WHO. Additionally, Walvax Biotechnology products have received regulatory approval. Two quadrivalent HPV vaccines are currently in Phase 3 clinical development. A well-phased multi-age cohort (MAC) campaign and the country’s willingness to accept all HPV vaccines will minimize the risk of shortages and significantly impact the long-term outlook for HPV vaccine supply (46, 81). Overall, five vaccines have been approved for marketing and WHO prequalification: 1) Three bivalent (HPV2) vaccines: 1.1) GSK’s Cervarix^®^ with proprietary AS04 adjuvant, indicated for girls and women, boys and men between 9-45 years of age. 1.2) Innovax’s Cecolin^®^ with aluminum-containing adjuvant, indicated for girls and women aged 9-45. 1.3) Walvax Biotechnology’s product with aluminum-containing adjuvant, indicated for girls and women aged 9-30 years (developed by its subsidiary Shanghai Zerun Biotech), 2) One quadrivalent (HPV4) vaccine: Merck’s Gardasil^®^ with aluminum-containing adjuvant, indicated from 9-45 years of age for girls and women, boys and men, 3) one nonavalent (HPV9) vaccine: Merck’s Gardasil 9^®^ with aluminum-containing adjuvant, indicated from 9-45 years of age for girls and women, boys and men (46). According to current public health evidence, the bivalent, quadrivalent, and monovalent vaccines show comparable immunogenicity, efficacy, and effectiveness in preventing cervical cancer (82). Regarding vaccine prices, GSK’s HPV2 product is $10.25 to $14.14 lower priced than Merck’s HPV4 product and $13.18 to $64.16 for self-procuring MICs. Prices have generally fallen across all sourcing and income groups over the past five years. If there is a reasonable demand for these new products, further price reductions will occur as future new entrants create a more competitive environment (46, 81).

### 2.3 Advocacy communications and social mobilization strategies

The introduction of HPV vaccination is an essential issue because juvenile girls are specifically targeted for HPV vaccination, and few vaccines have traditionally been suggested to adolescents in most states. From 2006 to 2010, the nonprofit global health institution PATH cooperated with some countries to help local managers decide how to present HPV vaccination, school-based and in health centers or a combination of both (56, 83). By strengthening positive stimuli for cancer prevention, health, welfare, and understanding vaccines as a robust public health intervention, community acceptance, and immunity coverage can be expanded (84). Governments have used various communication media to raise awareness and motivate girls, their families, and effective community concierges (85). Generally, girls’ most common sources of knowledge are doctors and nurses, family, friends, teachers, and neighbors. Other frequently used information channels are instant messaging programs, Internet search engines, community health workers, and social media. In addition, there are less popular sources, including traditional healers and mass media such as news channels, television programs, and newspapers (86). Therefore, social mobilization is required to increase the acceptance of HPV vaccination among girls, parents, and respected influencers. In other words, social acceptance of the value of HPV vaccination is crucial to coverage and the high effectiveness of vaccination. Social mobilization activities are vital to counter rumors and misinformation about HPV vaccination by raising awareness, providing accurate information, creating acceptance, and maintaining demand for HPV vaccination. So far, different strategies and materials have been used to contact the target audience. Methods of spreading information range from face-to-face gatherings in classrooms or clinics to in-home visits from health professionals to passive methods like handouts and broadcasts. Experience has shown that interactive approaches lead to a higher first dose coverage than non-interactive approaches alone. In contrast, interactive approaches were reported to be more successful than non-interactive ones. To influence the absorption of HPV vaccination, some governments have used combined methods (78). Crisis response strategies and authorized spokespersons have been established as a result of the widespread dialogue surrounding the HPV vaccine and other successful programs. But plans that included checking the media, especially social media, for false information regarding the HPV vaccine’s potential impacts on fertility and its harmful side effects were more successful at preventing rumors from hindering the vaccine delivery (32, 78). Surveys equip helpful feedback on which contact channels were most efficacious. Since this element is one of the expensive factors of a pilot program, such guidelines may be used in the future to design cost-effective social mobilization plans. Several governments have informed that printed items are often available on time or in insufficient quantities, but there is little proof that this impedes vaccine intake (84, 87). In total, community mobilization activities are practical when performed at least one month before vaccination using multiple pathways (88).

## 3 Ongoing challenges

Although many LMICs have succeeded in reaching substantial numbers of eligible girls, further progress is needed to overcome rumors, complete vaccine series, estimate the target population, monitor program performance, and ensure their sustainability. In general, the resolution of some issues is still unclear and needs further investigation (32). LMICs lack effective prevention and treatment of HPV-related diseases such as regular screening, public health policies, follow-up care, public education, and awareness raising (89). Thus, an incomplete understanding of eligibility criteria may lead to a loss of chance of vaccination or out-of-target vaccination. For example, Zimbabwe was challenged in identifying eligible people in its first year of vaccine introduction. In Zimbabwe, the challenges of the HPV vaccination program were pinpointed in general, including low social mobilization effort, poor personnel transport, and inadequate tools and training. The top three challenges informed by healthcare professionals were the inadequate transportation of staff and supplies, insufficient funding to provide vaccines, and the lack of a professional team to accomplish immunization (54). Also, there have been rumors of an increase in mortality rates among people vaccinated against HPV, while no vaccine-related deaths have been reported so far (61). Besides, special economic conditions, such as severe droughts and hurricanes, leading to rising commodity prices, limited fuel availability, reduced access to electricity and water, and raised food insecurity. They can be another key challenge in executing the HPV vaccination schedule (54). Moreover, parents’ skepticism about the HPV vaccine is the most likely obstacle to the successful implementation of the vaccination program in the community (86). It is clear that infrastructure issues, including supply shortages and staffing, have reduced access to medical services (90). Lack of proper identification of eligible girls is another challenge on the way to HPV vaccination, which can lead to missed vaccination opportunities, out-of-target vaccination, inaccurate registration and reporting, and ultimately inaccurate coverage (54). It is also difficult in many nations to get an accurate census of the number of out-of-school females and to know where they are located (88). Immunizing HIV-positive girls with the three recommended doses of HPV vaccine is another significant challenge, partly because of the close link between HPV vaccination interventions and prevention programs for HIV (91). Other restrictions on school immunizations may involve rumors and community or staff reluctance. School administrators can also create barriers by requiring written consent procedures and, in some cases, prohibiting vaccinations on school grounds (91, 92). Despite some challenges in dealing with rumors and gaining parental consent, experience has shown effective social mobilization and high uptake of the HPV vaccine in the LMIC (78).

### 3.1 Countermeasures for fighting rumors and mistrusts

A fundamental message has been determined based on previous experiences with the demo projects, focusing on cancer prevention, vaccine safety, government approval, clear explanations of immunization eligibility and the number of doses required, and practical information such as location and vaccination timing (85). Various factors affect the government’s ability to notify the public and the social atmosphere in which vaccination occurs. Most countries now have a good understanding of needed messages, but some barriers lead to a lack of timely distribution of materials or payments. Skepticism about government messages, sensitivity to rumors, and false information increased among ethnically diverse groups and other marginalized people. In this regard, historical or political reasons can contribute to government distrust. Many countries involve religious leaders in their planning, but opponent occurs for various religious reasons. Reporters sometimes have little knowledge or may be motivated to create or reinforce exciting stories about the apparent side effects of the vaccine. Anti-vaccination groups in other countries are adept at spreading misinformation swiftly *via* social media (32). A recent study in Malawi indicated that younger ages and lower education were risk factors for believing rumors about HPV vaccination, while trust in healthcare workers was a protective factor (93). In addition, a recent survey in Zimbabwe around the vaccination of multiple age cohorts revealed that despite considerable vaccine acceptance among participants, 42% of them were exposed to rumors around HPV vaccination safety and its side effects, including infertility threatening the vaccination uptake (54). As a whole, the center of the most convincing arguments on promoting awareness of cancer prevention measures, the security of vaccinations, and the widespread acceptance of these measures around the world. Also, community support should be increased, and negative rumors and publicity should be reduced (88).

### 3.2 Minimizing dropouts and treatment inequities

Numerous reasons have been cited for the failure of HPV vaccination, including poor follow-up systems, lack of continued social mobilization endeavors, insufficient training to complete subsequent vaccination courses, and transfer of girls to other schools. Therefore, these factors may lead to the failure of the annual vaccination schedules and receiving booster doses (16, 32). Due to the existing problems and obstacles, eliminating cervical cancer requires a substantial increase in current global health investments by revising spending priorities. In addition, while the link between HIV and HPV infection is well known, investment in HIV prevention needs to be accelerated to increase access to the HPV vaccination. In the short term, countries can benefit from international assistance to launch the HPV vaccination programs, but in the long run, they will need to establish domestic financial solutions to ensure the vaccine’s continued success (91). Despite achieving the highest coverage rates for the second dose, the school-based strategy has the highest cost of service delivery per dose compared to outreach or facility-based immunization. From a sustainable perspective, countries will incur higher costs for school delivery without the existing infrastructure to deliver school services. Governments must encourage a long-term vision for investing in school health programs to advance a wide range of adolescent health interventions, including HPV vaccination (87). Therefore, advocacy efforts to incentivize national planners to make adequate and appropriate investments to achieve and maintain high levels of coverage should be prioritized (91). Also, robust data systems are needed to identify eligible individuals better and use census and school data for micro-planning and evaluation of progress. Lacking in many nations are procedures that keep track of statistics, administer vaccinations, and serve as appropriate reminders. Investing in teacher training and health care providers for more active participation in information systems can better apply eligibility criteria. Mechanisms for enumerating, estimating, and monitoring the care of out-of-school girls are critical subjects for equity, as out-of-school girls are more vulnerable to health inequalities. Significant progress may be achieved by increasing the rate at which eligible girls, especially those who are not already enrolled in school, are enrolled to track them for their second dosage. A recent analysis of the Gavi-supported demonstration projects in 2017 indicated that there is a substantial dropout between the first and second doses and also between the second and third doses in twelve LMICs. The dropout rate was superior in facility-based programs (11%) compared to school-based delivery (7%) between first and second doses (87). In parallel, an investigation into immunization coverage and the dropout rate among Brazilian adolescent girls and their parents in 2020 demonstrated that there was a high vaccination dropout rate of 32.3% in the vaccination program. Given this considerable rate, the study underscores the role of educational intervention in raising awareness for vaccination in low-resource settings (94). Expanding 90% of HPV vaccine coverage by 2030 to multiple forces at all levels of operations, including support, communication, and participation, is essential to address the challenges, investment, and acceptance of the HPV vaccine (95). In addition, the increasing focus of international policymakers on the potential Covid-19 vaccine has been accompanied by a reduction in government funding due to the financial impact of the epidemic and the risk of slowing down efforts to increase access to the HPV vaccine at LIC/LMIC. Therefore, plans should be considered to provide that investments and efforts prevent cervical cancer, including HPV vaccination, continue during the epidemic and beyond (91). In this regard, facility-based schedules for families to send girls to immunization clinics are much cheaper than transmitting teams to schools. Still, more money is needed to inform and encourage girls to get regular vaccinations (85). From the side, building a high level of trust among the girl’s family, program leaders, and other stakeholders is essential to the immunization program’s success (86).

### 3.3 Multisectoral risk communication

Coordination between the health and education sectors is critical for most school-based programs. Still, even within the health sector, numerous separate divisions must collaborate to coordinate timetables and messages. Most countries appear to have a manageable system in place for training teachers, informing parents, and scheduling sessions around school exams and holidays(ref.). However, many countries still struggle with enumerating eligible girls, obtaining consent when necessary, identifying eligible girls if age is a criterion, and keeping records. Private schools can be more challenging to deal with and less willing to engage than public schools, and they may have stricter consent requirements. To develop effective remedies, more data and case studies are required (32, 44).

### 3.4 Securing political will, finances, and cultural acceptance

The effect of the on-time conduct of HPV vaccine in LMIC will be significant because every five-year deferment in supply leads to the death of 2 million people with cervical cancer (96). Therefore, the stability of HPV vaccine schedules was recognized as a cross-sectional challenge. Studies from separate countries determined obstacles to HPV vaccine stability (Bolivia, Rwanda, Cambodia, Haiti, Peru, Malaysia, Indonesia, Bhutan, Lesotho, Kenya, Vietnam, Uganda, India, Ghana, Tanzania, and Nepal) and broad world areas. The literature assessment identified financial, sociocultural, health, and political hurdles as the top three primary impediments to the implementation of HPV vaccination in LMICs.

Prior research from HICs and theoretical acceptance research in LMICs both predicted that sociocultural cases would be the greatest barrier to vaccine schedules; however, purposeful sensitization schedules have been victorious in overwhelming this challenge and reaching high vaccine acceptability covering levels in several LMICs (97).

Health system financial obstacles may disable the capability of some LMICs to effective implementation of high-quality HPV vaccine schedules. Securing endurable financing for the vaccine such as health system charges and holding donation schedules, represents a substantial barrier. A record low cost for as small as US $4.50 per dose for LICs, in comparison with over $100 in HICs, was reported in May 2013, by the GAVI Alliance (97). In other words, the price of the vaccine is one of the most crucial and major obstacles to the ultimate sustainability of the vaccine, whether a nation bears just a part of a Gavi-subsidized vaccine, a Gavi or PAHO-guaranteed cost, or the entire market cost. Budgeting for HPV vaccine at current prices presents a significant difficulty for governments with large populations, particularly those with middle incomes but no access to Gavi or PAHO expenditures (45).

The vaccine also introduces new political points, especially challenging the harmony of numerous stakeholder groups to maintain the motivation for supporting vaccination campaigns. The influence of political engagement in maintaining the integrity of vaccination campaigns is reflected in Rwanda’s successful HPV vaccination program, which reached over 93% of vaccine coverage after its first three-dose rollout in 2011. The success was attributed to the government agreement with Merck Company for providing the first three-dose for free, a population sensitization program powered by local government officials and healthcare professionals, and the development of technical working groups with other ministries to design a school-based delivery program (98). Thus it seems to gain the necessary political support for expanding the vaccination, solid local proof and education about cervical cancer will be crucial (97). Admission among society and prompt response to rumors must be maintained as these can derail vaccine schedules. In light of the experiences of Japan, India, and Denmark, the international society must remain alert and display contents to control rumors or parts of psychogenic diseases impacting delivery in the LIC/LMIC (45).

Significant progress has been accomplished in many LMICs, and it is crucial to understand lessons that are transferable to other settings. While school-based schedules have been successful, innovative sensitization and delivery strategies will be required to ensure equitable distribution. Demonstration and experimental schemes in multiple LMICs have revealed that practices such as delivery *via* schools, particular campaigns, healthiness centers, or several techniques can achieve a considerable proportion of qualified girls and that support and sensitivity are essential. GAP nations such as Uganda, Lesotho, and Cameroon have lately communicated lessons from experimental measures such as the significance of gaining political commitment, mustering resources, and suggesting merging the vaccine within existing immunization networks for stability (97). An assessment of eight GAP pilots discovered that delivery *via* hybrid models in both schools and health centers was the most influential (56).

It is, however, argued that HPV vaccination programs need to be tailored to individual regions, and that successful demonstration schemes are not necessarily transferable to other LMICs. Previous experiences with HPV vaccination in low- and middle-income countries have had encouraging results, data, successes, and failures must be shared once again so that successful HPV vaccine programs can be replicated around the world (97). In 2018, around 80 nations worldwide had HPV vaccination schedules; the obstacles to HPV vaccine presentation had stayed most significant in those nations with the most cases of cervical cancer and the most demand for vaccination. Grant for qualified LIC/LMIC was accessible *via* Gavi to vaccinate 40 million girls by 2020; nonetheless, global commitment is required to keep existing HPV vaccine schedules and spread support after 2020. In the long term, labor is necessary to secure a sustainable structure of international grants and support health plans and immunization schedules until the HPV vaccine becomes just one platform of services provided to youthful males and females. In the short run, some of the most significant barriers to expanding the extent and sustained use of HPV vaccination in LIC/LMIC may be surmounted if a single dose of the vaccine proves effective (45). In this regard, Barnabas and colleagues showed during a 2022 study that the single-dose bivalent and nonavalent HPV vaccines were highly efficacious in preventing HPV infection. This finding could help close the gap between the WHO targets of 90% HPV vaccine coverage by 2030 and reduce vaccine supply constraints.

Besides health impacts, one of the notable aspects of the HPV vaccination program through vaccines with different valency and prices is their cost-effectiveness. The decision to incorporate more affordable vaccines versus more expensive but more effective ones has extra importance for LMIC governments (99). This way, economic modeling, and cost-effectiveness investigations could help mobilize resources toward sustainable options. One study in the Philippines revealed that among four available HPV vaccines (Cecolin, Cervarix, GARDASIL9, and GARDASIL), the more expensive and privately marketed GARDASIL9 was not a cost-effective option by GDP per capita. Compared to no vaccination, the other three vaccines were cost-effective and suitable for boosting the nation’s vaccination program, while Cecolin was the most sustainable in economic consideration (81).

Although economic modeling is one of the pieces of decision-making, the vaccine’s extra valency with its added price may not necessarily confer further benefits for the budget, and vaccine supply restrains in LMIC. The other factors engaged in the choice of vaccine introduction are the distribution of HPV variants and demographic factors related to vaccine approval (16). The importance of funding support for vaccine programs is also reflected in the analysis in Bangladesh with no HPV vaccination program, showing that bivalent Cervarix is cost-effective only at Gavi-provided prices. Neither Cervarix nor quadrivalent GARDASIL was cost-effective at market price and different delivery routes (51). This highlights the vaccine price as one of the dominating factors in adding more compliance to vaccine introduction in line with other studies in LMIC, including Kenya and Uganda (100).

### 3.5 Supply limitations of HPV vaccines

The main burden (86%) of cervical cancer is in LMICs, while < 30% of these nations have presented the vaccine. The condition is aggravated by limitations in the vaccine, supply possible to continue until 2022/25 (101). Also, LMICs can anticipate supply limitations to restrict their capacity to present the vaccine or perform MACs. Many countries that would have otherwise qualified for Gavi had to delay their MACs and shift their single-age cohorts to older girls as a result of this change in planning. In Tanzania, after a successful two-year pilot school-based vaccination program with quadrivalent vaccines initiated in 2014, the government faced global supply constraints for importing quadrivalent vaccines supported by Gavi. This insufficient supply forced the decision-makers to implement a single cohort vaccination program for 14-year-old girls and further expanded efforts to reach this single cohort until there was sufficient supply (102). This has an impact on non-Gavi countries as well because it limits their ability to negotiate reasonable prices when they compete with more developed countries that are implementing gender-neutral scheduling and serving a wider age range (32). In November 2019, WHO SAGE augmented the ongoing advantages of the programmatic innovation, such as maximizing service delivery efficiencies concerning global supply limitations, and suggests in the duration of supply limitation that the initial target population for HPV vaccination must persist in being girls aged 9 to 14 years, before starting sexual activity, with a two-dose program (91). SAGE has suggested temporarily stopping gender-neutral performance, older age (>15 years), and MACs vaccination as long as sufficient resources are available to handle demands worldwide reasonably. The discussion is to let these vaccine doses be utilized in governments seeking to begin an HPV vaccine schedule for girls or nations with restricted vaccine supply (103). Needed doses have also been compared using other situations, such as a 2-dose program vs a 1-dose program, with or without compensatory vaccination, and long-term programs where the second dose is delayed by either 3 or 5 years. In the short term, all scenarios experience a shortage at a foundational supply level, with the 3 years having the least significant effect. Yet, in the intermediate to long run, vaccine reserves will be sufficient. Nonetheless, only 1-dose programs will have sufficient supplies in the middle to long term, with a longer interval of 3 years as a potential replacement in the case of low supply (104). This will reduce supply constraints quickly and allow the assignment of doses to high-burden nations now scheduling to present this vaccine. Cecolin^®^, a vaccine against HPV, has obtained prequalification from the WHO. Cecolin is produced by Xiamen Innovax Biotech CO., LTD. (Innovax), a completely owned subsidiary of Beijing Wantai Biological Pharmaceutical Co., LTD. (Wantai), and is ready to defend against HPV types 16 and 18, the most usual virus types which lead to cervical cancer. Governments facing obstacles to national introduction or developing their HPV vaccine schedule to the whole cohort owing to cost or supply constraints will now have another choice for cost-effective, stable access. A summary of progress, ongoing challenges, and prospects of national HPV vaccination programs is presented in **Figure 3**.

## 4 Future directions

There are potential scientific and manufacturing improvements that could have a significant effect on future HPV vaccination schedules. Despite targeting a single cohort in specific periods that may sound more cost-saving for LMICs facing financial and technical obstacles, inbitiating multi-age cohorts may lead to more rapid and comprehensive immunization coverage among target groups (10). In addition, new and more economical vaccine sources could alter the situation, allowing males and older women to get vaccinated. In parallel, countries may have to examine trade-offs between price, efficacy, and impact when selecting vaccines if the features of future vaccines are significantly different from those of existing vaccines. Moreover, multiple clinical trials have shown encouraging results for one-dose regimen HPV vaccines, demonstrating comparable efficacy to current vaccines (1) that may partly overcome the current issues in the cost-effectiveness of vaccination programs in several LMICs. Besides, as it mentioned, based on the most recent investigations that has been published in December 2022, WHO recommends: A one or two-dose schedule for girls aged 9 to 14 year; A one or two-dose schedule for girls and women aged between 15 to 20 year; Two doses with a 6-month interval for women older than 21 years. Paying attention to these strategies, might be the most effective preventable strategy for cervical cancer increasing, HPV infection and cancer costs, and social burden of these diseases.

**Figure 5:**
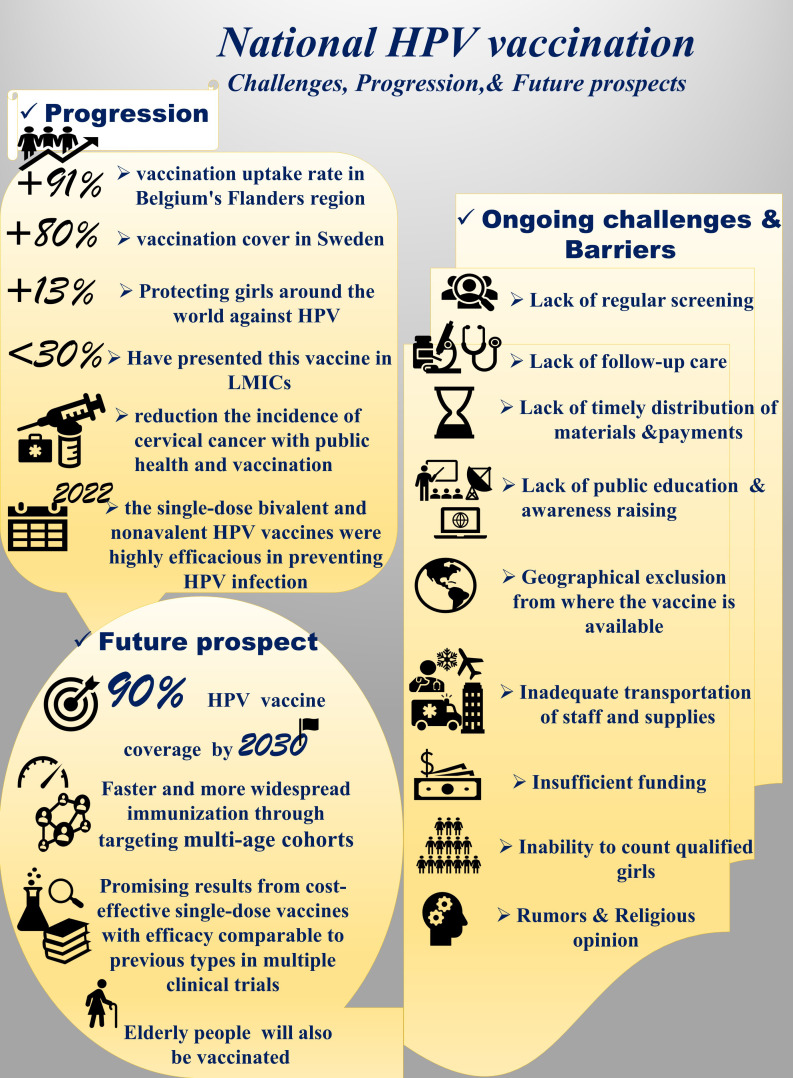
**A** summary of progress, ongoing challenges, and future prospects of national HPV vaccination programs.

A summary of progress, ongoing challenges, and future prospects of national HPV vaccination programs is presented in **Figure 5**.

## 5 Conclusions and recommendations

Various approaches can efficiently deliver the HPV vaccine to young adolescent girls. Gavi funding has been critical for covering the costs of vaccinations and early distribution in low and lower-middle-income countries. The possibility of new, more effective vaccine entry into the market may enable overcoming this barrier, while other issues or factors may be preventing some governments from doing so. The lessons learned from successful vaccination programs in HIC could guide health policymakers to take more proactive steps. Regarding the speed of vaccine introduction, LMIC should accelerate the process since they outnumber HIC, has more population, and have initiated the program later. Although current school-based programs have exhibited promising results in LMIC, the vaccine delivery route may also benefit from more engaging facility-based services compared to its dominant school-based form in LMIC (35). This is noteworthy because of concerns around the costs of school-based programs and the inequity to reach girls in regions with lower school enrolment (85). Due to less developed public infrastructure for cervical cancer screening and also for diagnosis and treatment, enhancing these facilities should be prioritized in conjugation with immunization schedules. This is especially important in sub-Saharan LMIC with the highest cervical cancer rates, for which vaccination alone is insufficient to eliminate HPV-related cancers. In a recent model, it was predicted that the goal of cancer elimination might be achieved in LMIC by reaching 90% uptake in vaccination and a twice-lifetime screening program by the end of the century (33). Given the inappropriate vaccine coverage issue in LMIC, motivating at-risk populations with financial support and better education on health information may elevate the chance of the HPV vaccination program’s success (16). Considering some negative effects of media and healthcare providers on vaccine acceptance and coverage experienced in HIC, LMIC should implement a surveillance system and counteractive measures against health rumors in the media and educational intervention for health providers to endorse vaccination awareness for the target cohort and their parents (105–107). The national introduction of the HPV vaccine is vital in reducing and eliminating cervical cancer. However, the use of previous skills and knowledge and progress on the frontier to increase stability and efficiency, as well as progress toward the coverage levels required for the eventual elimination of this cancer (108, 109).

In conclusion, several approaches should be considered. Educating and teaching people about compliance with public health, testing, and routine checkups in overcrowded places like pools, dormitories, schools, and even armies, teaching self protections in sex, making protected sex products available and reducing the cost of them, increasing screening tests, especially before marriage, mass vaccination, developing new effective vaccines in theire own country, and predicting appropriate treatment budgets for cervical cancer. On the other hand, WHO and high-income countries should regard supporting and increasing HPV vaccine availability for low-and middle-income countries. We must remember that if we neglect it, the next pandemic may be caused by HPV infection.

## Author contributions

NEb, ZY, GK, FE, MG, MA, MS, BG, and NEs conceptualized the study and wrote the manuscript. NEb, ZY, GK, FE, MG, and MA contributed to drafting of the manuscript. All authors contributed to the article and approved the submitted version.
